# Electroacupuncture Ameliorates Acute Pancreatitis: A Role for the Vagus Nerve–Mediated Cholinergic Anti-Inflammatory Pathway

**DOI:** 10.3389/fmolb.2021.647647

**Published:** 2021-05-13

**Authors:** Luyao Zhang, Zhiyang Wu, Jing Zhou, Shengfeng Lu, Chaofan Wang, Yiqiu Xia, Hongyan Ren, Zhihui Tong, Lu Ke, Weiqin Li

**Affiliations:** ^1^ Department of Pathology, School of Medicine and Holistic Integrative Medicine, Nanjing University of Chinese Medicine, Nanjing, China; ^2^ Department of Critical Care Medicine, Qilu Hospital (Qingdao), Cheeloo College of Medicine, Shandong University, Qingdao, China; ^3^ Department of Critical Care Medicine, Jinling Clinical Medical College of Southeast University, Nanjing, China; ^4^ Key Laboratory of Acupuncture and Medicine Research of Ministry of Education, Nanjing University of Chinese Medicine, Nanjing, China; ^5^ Department of Critical Care Medicine, Jinling Hospital, Medical School of Nanjing University, Nanjing, China

**Keywords:** electroacupuncture, pancreatitis, vagus nerve, α7 nicotinic acetylcholine receptor, anti-inflammation

## Abstract

Organ failure resulting from excessive inflammation is the leading cause of death in the early phase of acute pancreatitis (AP). The autonomic nervous system was reported to be involved in AP, and the vagus nerve could exert anti-inflammatory effects through α7 nicotinic acetylcholine receptor (α7nAChR) signaling. Acupuncture has been widely used in traditional Asian medicine, and recent studies suggested the inflammation modulating effect of electroacupuncture (EA) might be mediated by the autonomic nervous system. In this study, we aimed to investigate the effects of EA in AP animal models. Two independent AP mouse models were used, namely, caerulein hyperstimulation and pancreatic duct ligation. We found that EA at Zusanli acupoint increased vagus nerve activity, suppressed systemic inflammation, and alleviated the histopathological manifestations and leukocyte infiltrations of the pancreas. Induction of AP resulted in a remarkable decrease in the frequency of α7nAchR^+^ macrophages in the pancreas, while EA counteracted this phenomenon. The anti-inflammatory, pancreatic protective and upregulation of α7nAchR effects of EA were reduced in mice with vagotomy. Moreover, the therapeutic effects of EA were attenuated in mice treated with methyllycaconitine citrate, a selective α7nAChR antagonist. Taken together, EA could modulate inflammation, thereby exerting protective effects in AP. The mechanism may include activating the vagus nerve through the cholinergic anti-inflammatory pathway.

## Introduction

Acute pancreatitis (AP) is initiated with inappropriate activation of pancreatic enzymes and subsequent local and systemic inflammatory responses (Kang et al., 2014; Dixit et al., 2019). Inflammatory mediators released from injured acinar cells and immune cells mediate pancreatic injury and exacerbation of AP (Pandol et al., 2007; Zhang et al., 2013). Accordingly, several therapeutic approaches targeting inflammatory cytokines in AP have been proposed (Xue et al., 2014). However, none of them proved clinically effective.

In 2000, Kevin J. Tracy first described a parasympathetic anti-inflammatory pathway (Borovikova et al., 2000). Further, research studies found the anti-inflammation effect of vagus nerve stimulation was mediated by activation of macrophages *via* α7 nicotinic acetylcholine receptor (α7nAChR), which is called cholinergic anti-inflammatory pathways (Inoue et al., 2016; Fox, 2017; Nishio et al., 2017). However, direct vagus nerve stimulation needs invasive operation under general anesthesia, which limits its clinical use. Thus, a less invasive method for vagus nerve stimulation may be of clinical implication.

Acupuncture is a traditional Chinese practice with a core idea that stimulation at specific parts of the body (acupoints) can distantly modulate visceral organ physiology (Liu et al., 2020). Electroacupunctuture (EA) is a combination of acupuncture and electric stimulation applying electric current to pairs of acupuncture needles. Researchers found EA exerts immune regulatory effects in a series of inflammatory diseases (Torres-Rosas et al., 2014; Ma et al., 2017; Chi et al., 2018), but the underlying mechanisms remain unclear. Recent findings have highlighted a key role for autonomic nerve activity in mediating the anti-inflammatory signaling of EA, indicating EA can be an alternative for vagal stimulation (Liu et al., 2020).

In this study, we aimed to evaluate the effects of EA at Zusanli (ST-36) acupoint in experimental AP and investigate the specific role of the vagus nerve and α7nAChR on macrophages.

## Material and Methods

### Mice

Wild-type mice (ICR; male; 25–30 g) were purchased from the Qing Longshan Animal Breeding Facility (Jiangning, Nanjing, China). Mice were maintained under specific pathogen-free conditions in accordance with the National Institutes of Health Guide for the Care and Use of Laboratory Animals. All animal protocols were reviewed and approved by the Institutional Animal Care and Use Committee of Nanjing University of Chinese Medicine and were performed in accordance with the guidelines for animal research.

### Experimental Acute Pancreatitis

A mild edematous pancreatitis was induced in mice by 10 hourly intraperitoneal injections (i.p.) of 100 μg/kg caerulein (NJPeptide, Inc., Nanjing, China). The normal control (NC) group received PBS. Two hours following the last injection, mice were sacrificed under general anesthesia, and their blood was collected from the vena cava inferior in heparin-coated vacutainer tubes. The pancreas was removed for flow cytometry and histological analysis. The lung was removed for immunohistochemistry.

The pancreatic duct ligation (PDL) model was used to mimic severe gallstone-induced pancreatitis as described by Samuel (Samuel et al., 2010). Briefly, mice were anesthetized under inhaled isoflurane (2% induction and 1.5% maintenance with spontaneous respiration, in 30% O_2_). The duodenum was exposed, and the distal common bile-pancreatic duct was ligated near its junction with the duodenum. Then, the abdominal cavity was closed, and the mice were placed on a heating table with a constant temperature of 37°C during recovery. Mice were sacrificed, and samples were collected 48 h after PDL modeling.

### Electroacupuncture Stimulation Technique

EA was performed under inhaled isoflurane by stimulating both limbs at the ST-36 Zusanli acupoint. The ST-36 Zusanli acupoint is located 2 mm lateral to the anterior tubercle of the tibia in the anterior tibial muscle and 4 mm away from the knee joint lower point (Torres-Rosas et al., 2014). Unipolar stainless steel needles were inserted about 3 mm deep into the ST-36 acupoint. The acupoints were then stimulated with an electrical stimulator (200A, HANS, Nanjing, China). Electrical stimulation (current, 2 mA; frequency, 2/15 Hz) was applied for 20 min long. Mice in sham groups were treated by a needle inserted at the ST-36 acupoint without an electrical current. In caerulein AP mice, EA was applied immediately after the first caerulein injection. In PDL AP mice, EA was applied 30 min and 24 h after operation.

### Vagotomy

Mice were anesthetized with inhaled isoflurane and subjected to left cervical vagotomy or sham operation 3 days before pancreatitis induction. Briefly, a ventral cervical incision was made, and the left vagus nerve was exposed. Vagus nerve truck was ligated with 4-0 silk sutures and transected. Subsequently, the skin was closed. In sham-operated animals, the left cervical vagus nerve was exposed but not transected.

### Heart Rate Variability Analysis

A 5-min electrocardiographic signal was obtained from isoflurane-anesthetized mice immediately after EA by an electrocardiogram machine (EDAN, Shenzhen, China). Normal beat intervals were identified from the lead II ECG recordings and analyzed using the frequency domain analysis. The power spectrum of the beat-to-beat intervals was generated using fast Fourier transformation. The low frequency/high frequency (LF/HF), which was used as the index of sympatho-vagal balance, was calculated (Author Anonymous, 1996). Elevation in LF/HF shows sympathetic predominance, while decrease of LF/HF indicates parasympathetic predominance.

### Plasma Amylase and TNF-α, IL-1β, and IL-6 Detections

Levels of amylase in the plasma were measured with commercially available kits (BioSino Bio-Technology and Science Inc., Beijing, China) according to the manufacturer’s instructions. Results are expressed in international units (U) per liter (L). TNF-α, IL-1β, and IL-6 levels in the plasma were analyzed by ELISA kits (MultiSciences, Hangzhou, China).

### Histological Evaluations of the Pancreas

The pancreas was harvested, fixed in neutral-buffered formaldehyde, embedded in paraffin, and sectioned and stained with H&E. All specimens were scored blindly by a pathologist unaware of the groups. Pancreas damage was scored based on necrosis, inflammation, hemorrhage, and edema (0–4 scale each) as previously described (Schmidt et al., 1992).

### Isolation of Pancreatic Acinar Cells and Leukocytes and Flow Cytometry

Pancreatic acinar cells and leukocytes were obtained using a collagenase digestion method described previously for flow cytometry analysis (Wu et al., 2018). The pancreas was removed, minced into small fragments, and digested in a DMEM medium supplemented with 2% fetal bovine serum and collagenase IV (BioFroxx, Germany, at a concentration of 2 mg/ml). Samples were incubated under agitation for 15 min at 37°C and vortexed at a low speed for 20 s before passing through a 70-mm filter. Pancreas homogenates were subjected to centrifugation to obtain pancreas acinar cells and leukocytes.

Pancreatic leukocytes were stained with the following Abs: anti-CD11b APC/Cy7 (clone M1/70, Biolegend, San Diego, CA, United States), anti-Ly6G FITC (clone RB6-8C5, eBioscience, CA, United States), anti-F4/80 PE/Cy7 (clone BM8, Biolegend, San Diego, CA, United States), and anti-α7nAChR PE (clone 319, Santa Cruz). Cells were suspended in a Pharmingen Stain Buffer and analyzed using a ACEA NovoCyte flow cytometer with NoVo Express software (ACEA Biosciences, San Diego, CA, United States).

### Immunohistochemistry for MPO

After de-waxing and rehydrating, the lung sections were heated for antigen retrieval and washed with 3% H_2_O_2_ to remove endogenous peroxidase activities. Then, the lung sections were incubated with primary antibodies against myeloperoxidase (MPO, Abcam, 1:100 dilution) at 4°C overnight. The secondary antibody was incubated for 1 h at room temperature.

### Statistical Analysis

All data are expressed as means ± SEM. Comparisons between groups were made using one-way ANOVA followed by the Turkey test. Histological and immunohistochemistry scores were analyzed with the Kruskal–Wallis test followed by Dunn’s test. Survival curves were derived by the Kaplan–Meier method and compared by the log-rank test. A *p* value<0.05 was considered significant. All the analyses were performed with GraphPad Prism version 7 (GraphPad software).

## Results

### Electroacupuncture Enhanced Cardiac Parasympathetic Activity in Caerulein Acute Pancreatitis Mice

**FIGURE 1:**
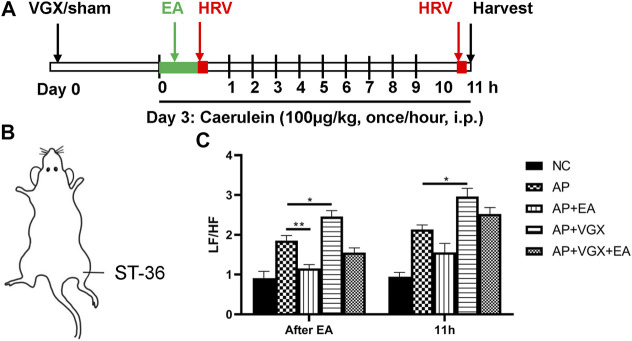
Effects of EA on HRV in caerulein AP mice. **(A)** Schematic description of study design. Vagotomy/sham operation was performed three days before model induction. EA was applied immediately after the first caerulein injection, and HRV was obtained afterward. Mice were sacrificed 2 hours after the last caerulein. **(B)** The location of ST-36 in mice. **(C)** The LF/HF of HRV after EA treatment and at 11 h later. *n* = 7–10. Bars show mean ± SEM. *p < 0.05, **p < 0.01. VGX, vagotomy; EA, electroacupuncture; HRV, heart rate variability; i.p., intraperitoneal injection; NC, normal control; AP, acute pancreatitis; LF/HF, low frequency/high frequency.

To determine the therapeutic effects of EA, caerulein hyperstimulation–induced AP mice were used (Figure 1A). Mice were subjected to vagotomy 3 days before AP induction. EA at ST-36 (Figure 1B) was applied immediately after the first caerulein injection, and HRV was obtained right afterward and at 11 h later. The plasma and the pancreas were harvested at the 11th hour. As shown in Figure 1C, the LF/HF measured immediately after EA was significantly lower in the EA group than in the AP group, indicating EA treatment resulted in parasympathetic predominance. However, no statistical difference was found in the AP group and the AP + EA group at the 11th hour. As expected, vagotomy increased LF/HF at both time points.

### Effect of Electroacupuncture on the Severity of Caerulein-Induced Pancreatitis

**FIGURE 2:**
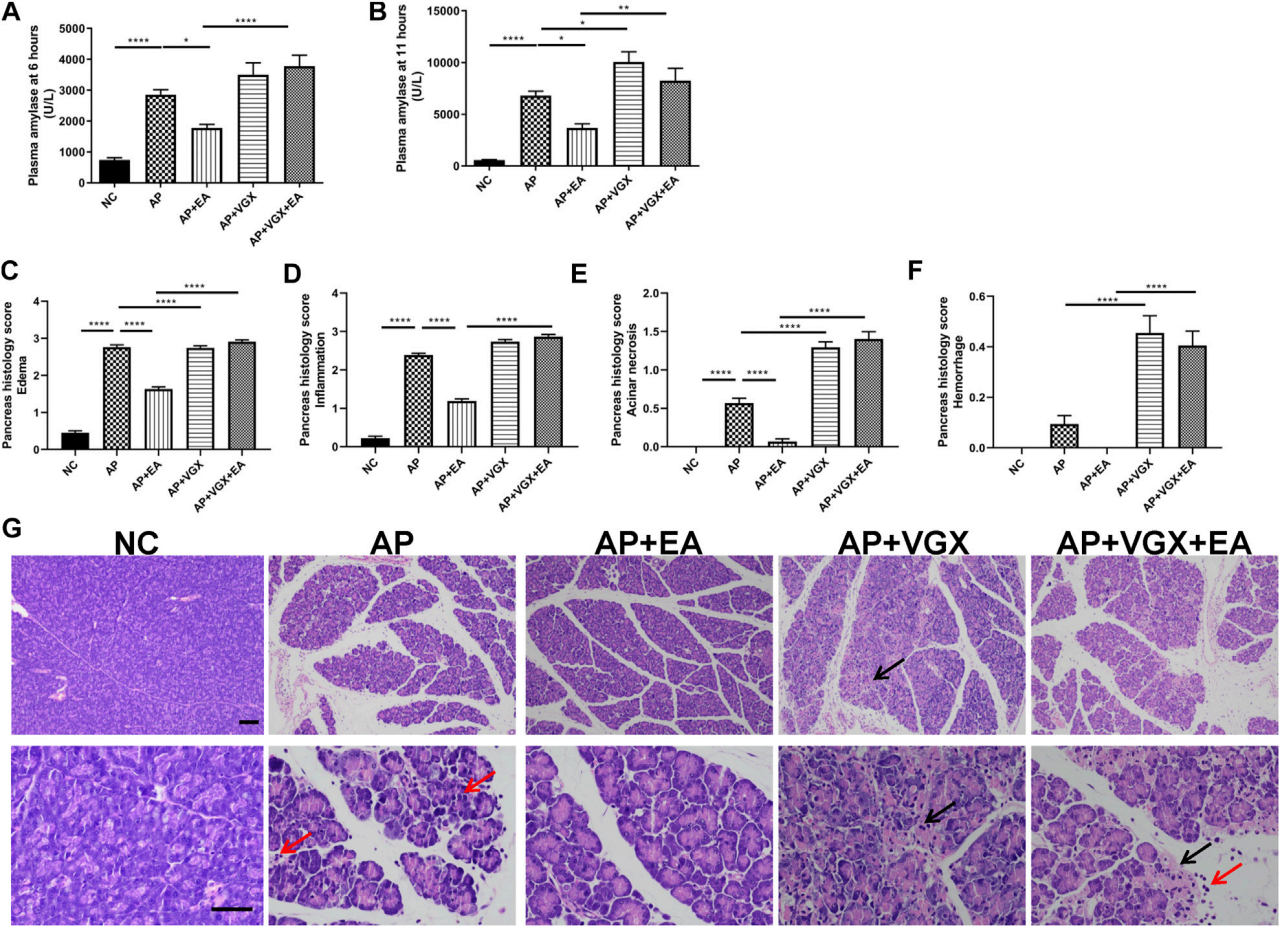
EA alleviates the severity of caerulein AP mice. **(A)** Plasma amylase activity at 6 h after the first caerulein injection. **(B)** Plasma amylase activity at 11 h. **(C)** The pancreas histological score of edema. **(D)** The pancreas histological score of inflammation. **(E)** The pancreas histological score of acinar necrosis. **(F)** The pancreas histological score of hemorrhage. **(G)** Representative H&E staining of pancreas sections (scale bars: 100 μm). Black arrow represents pancreas necrosis, and red arrow represents infiltrating cells, *n* = 7–10. Bars show mean ± SEM. *p < 0.05, **p < 0.01, and ****p < 0.0001. NC, normal control; AP, acute pancreatitis; EA, electroacupuncture; VGX, vagotomy.

Some parameters which characterize the severity of pancreatitis were measured. Low levels of plasma amylase activities were evidenced in the NC group (Figures 2A,B). Caerulein increased plasma amylase levels to 2857 ± 162.1 U/L at 6 h and 6813 ± 433.5 U/L at the 11th hour after the first caerulein injections. EA treatment showed marked reductions of amylase levels at both time points. Eleven hours after the first caerulein injection, histological examination of the pancreas showed severe edema and massive leukocytes infiltrations in AP mice (Figures 2C–G). EA treatment of AP mice also significantly lowered the histological manifestation of the disease. Pancreatic edema, inflammation, and acinar necrosis histological evaluations were remarkably improved in EA mice compared with AP mice. Furthermore, vagotomy exaggerated pancreatitis severity. The plasma amylase concentration at 11 h increased in vagotomy mice as compared with AP mice. Intensive inflammatory cell infiltration and focal areas of necrosis were found in the pancreas of vagotomy mice. Cervical vagotomy dampened the protective effects of the EA. No statistical difference was found in plasma amylase concentrations and pancreatic histological injury between vagotomy mice and vagotomy plus EA treatment mice.

**FIGURE 3:**
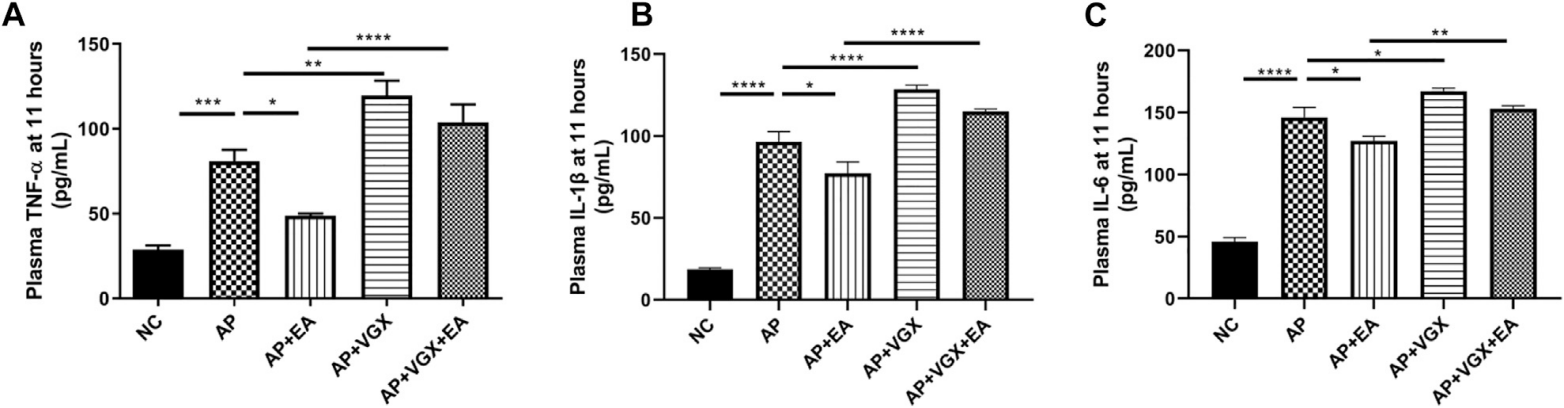
EA decreases systemic cytokines in caerulein AP mice. **(A)** Plasma TNF-*α* levels at 11 h after the first caerulein injection. **(B)** Plasma IL-1β levels at 11 h. **(C)** Plasma IL-6 levels at 11 h, *n* = 7–10. Bars show mean ± SEM. *p < 0.05, **p < 0.01, ***p < 0.001, and ****p < 0.0001. NC, normal control; AP, acute pancreatitis; EA, electroacupuncture; VGX, vagotomy.

Systemic inflammatory cytokines play a central role in the progression of severe AP. Next, we investigated TNF-α, IL-1β, and IL-6, the main mediators of the acute phase response whose levels are useful for predicting the severity of AP (Rodriguez-Nicolas et al., 2018). EA treatment significantly decreased plasma TNF-α, IL-1β, and IL-6 levels in AP mice (Figure 3). Conversely, vagotomy increased all these three cytokine levels, and no beneficial effect of EA treatment was found in the absence of an intact vagus nerve.

### Effect of Electroacupuncture on Infiltration of Macrophages, Neutrophils, and α7nAChR^+^ Macrophages in the Pancreas in Caerulein Acute Pancreatitis Mice

**FIGURE 4:**
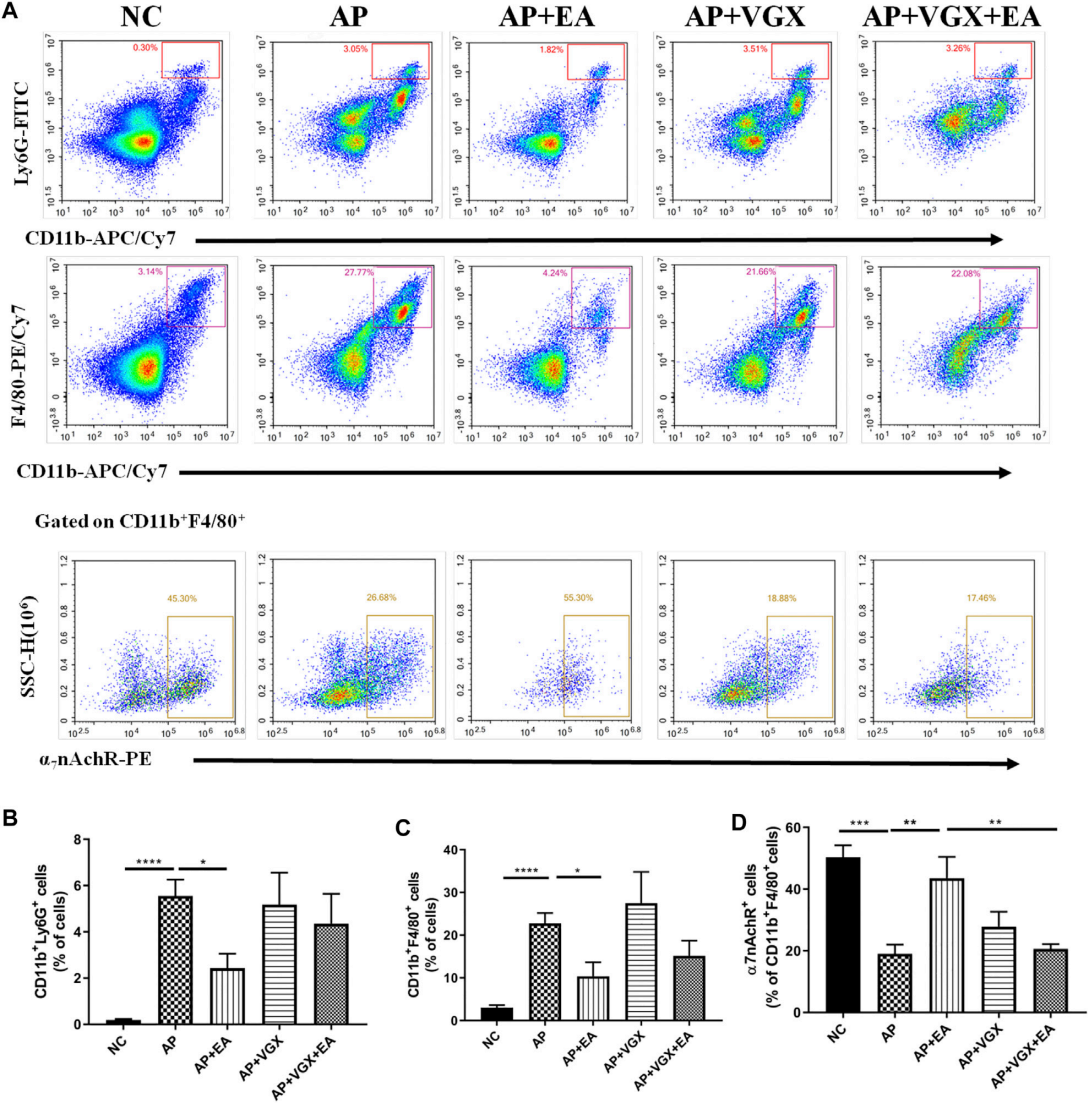
EA decreases the infiltrations of neutrophils and macrophages and increases the frequency of α7nAChR^+^ macrophages in the pancreas of caerulein AP mice. Pancreas-infiltrating leukocytes and acinar cells were isolated for flow cytometry **(A)** and percentages of CD11b^+^Ly6G^+^ neutrophils **(B)** and CD11b^+^F4/80^+^ macrophages **(C)** were analyzed. The percentage of α7nAChR-positive macrophages was also analyzed **(D)**, *n* = 7–10. Bars show mean ± SEM. *p < 0.05, **p < 0.01, ***p < 0.001, and ****p < 0.0001. NC, normal control; AP, acute pancreatitis; EA, electroacupuncture; VGX, vagotomy.

Neutrophils and macrophages, recruited into the pancreas tissue in the early phase of AP, are critical in the pathogenesis of AP (Habtezion, 2015). We next investigated these two cell populations affected by EA. As shown in Figures 4A,B, there were significant influxes of neutrophils (CD11b^+^Ly6G^+^ cells) and macrophages (CD11b^+^F4/80^+^) into the pancreas after induction of AP (*p* < 0.001). EA-treated mice exhibited a significantly lower percentage of neutrophils (2.4 ± 0.6% *vs.* 5.5 ± 0.7%, *p* < 0.05) and macrophages (10.4 ± 3.3% *vs.* 22.7 ± 2.5%, *p* < 0.05) in the pancreas in comparison to AP mice. However, vagotomy had no impact on the percentages of neutrophils and macrophages (Figures 4B,C) in AP mice.

We further examined the expression of α7nAChR on macrophages. In the NC mice, 50.3 ± 3.9% macrophages expressed α7nAChR, and only 19.3 ± 3.0% α7nAChR^+^ macrophages were found in the AP mice (Figure 4D). EA-treated mice displayed a distinct higher frequency of α7nAChR^+^ macrophages (43.6 ± 6.9%) compared with the AP group (*p* < 0.01). Also, the effect of EA on the frequency of α7nAChR^+^ macrophages was abolished in mice pretreated with vagotomy.

### Blockade of α7 Nicotinic Acetylcholine Receptor Reduced the Protective Effect of Electroacupuncture on Caerulein-Induced Pancreatitis

**FIGURE 5:**
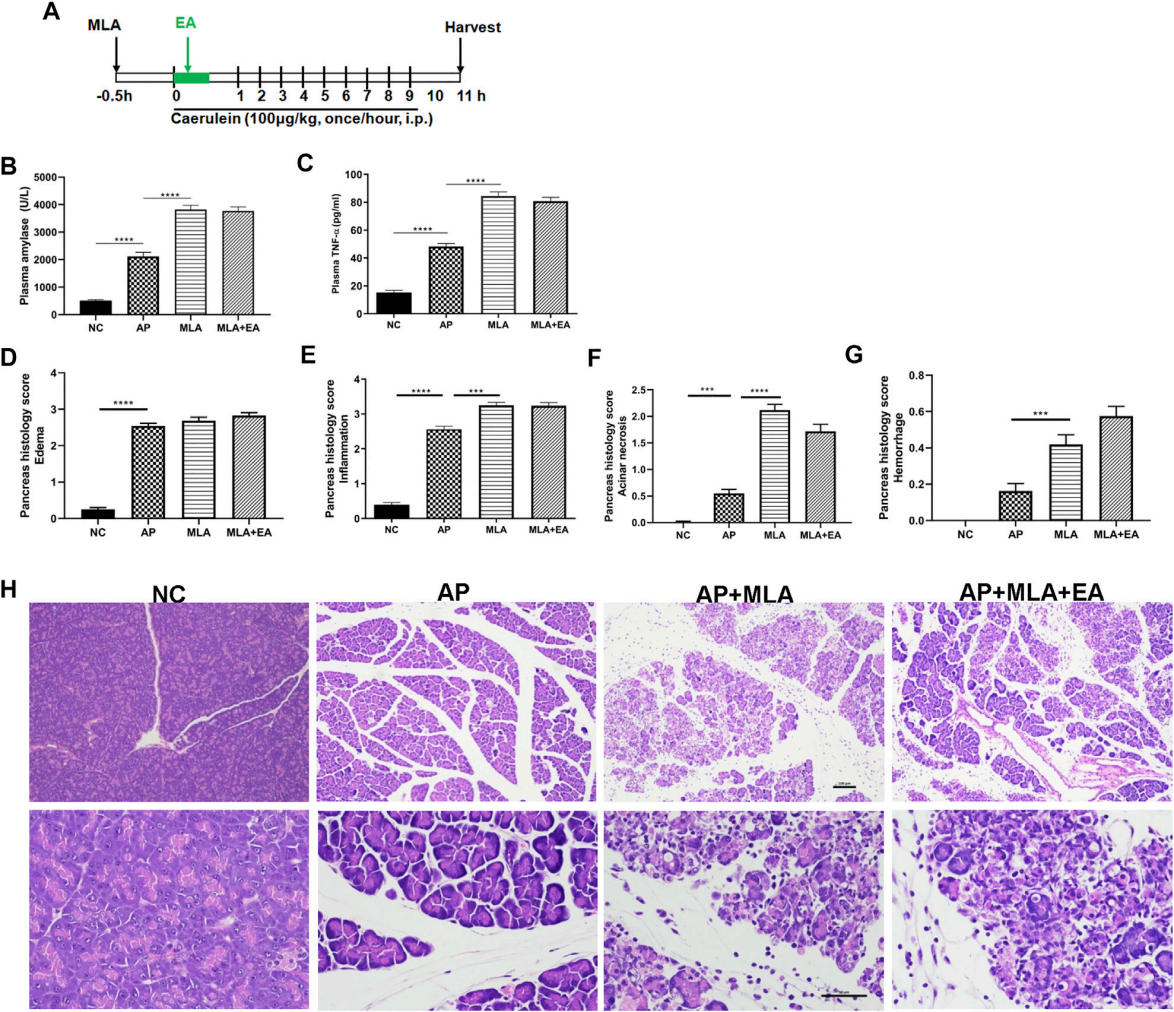
The effects of EA in caerulein AP mice are mediated through α7nAChR. Methyllycaconitine citrate (MLA, a selective α7nAChR antagonist, 3 mg/kg, i.p.) was administered 30 min before the first caerulein injection. **(A)** Schematic description of time line. **(B)** Plasma amylase activity at 11 h after the first caerulein injection. **(C)** Plasma TNF-*α* at 11 h. **(D)** The pancreas histological score of edema. **(E)** The pancreas histological score of inflammation. **(F)** The pancreas histological score of acinar necrosis. **(G)** The pancreas histological score of hemorrhage. **(H)** Representative H&E staining of pancreas sections (scale bars: 100 μm), *n* = 8. Bars show mean ± SEM. ***p < 0.001 and ****p < 0.0001. MLA, methyllycaconitine citrate; NC, normal control; AP, acute pancreatitis; EA, electroacupuncture.

Having established that EA increased the frequency of α7nAChR^+^ macrophages in the pancreas, we next set out to determine whether pharmacologic blockade of α7nAChR is required for the protective effect of EA. Caerulein pancreatitis was induced half an hour after i.p. with methyllycaconitine citrate (Figure 5A). Pretreatment with methyllycaconitine citrate (3 mg/kg) dampened the suppressive EA effect on pancreatitis development. No significant differences were detected in plasma amylase and TNF-α levels (Figures 5B,C) and pancreas histology evaluation (Figures 5D–H). These results indicate α7nAChR is essential for the alleviation of pancreatitis by EA.

### Effect of Electroacupuncture on Disease Severity and the Survival Rate in Pancreatic Duct Ligation Mice

**FIGURE 6:**
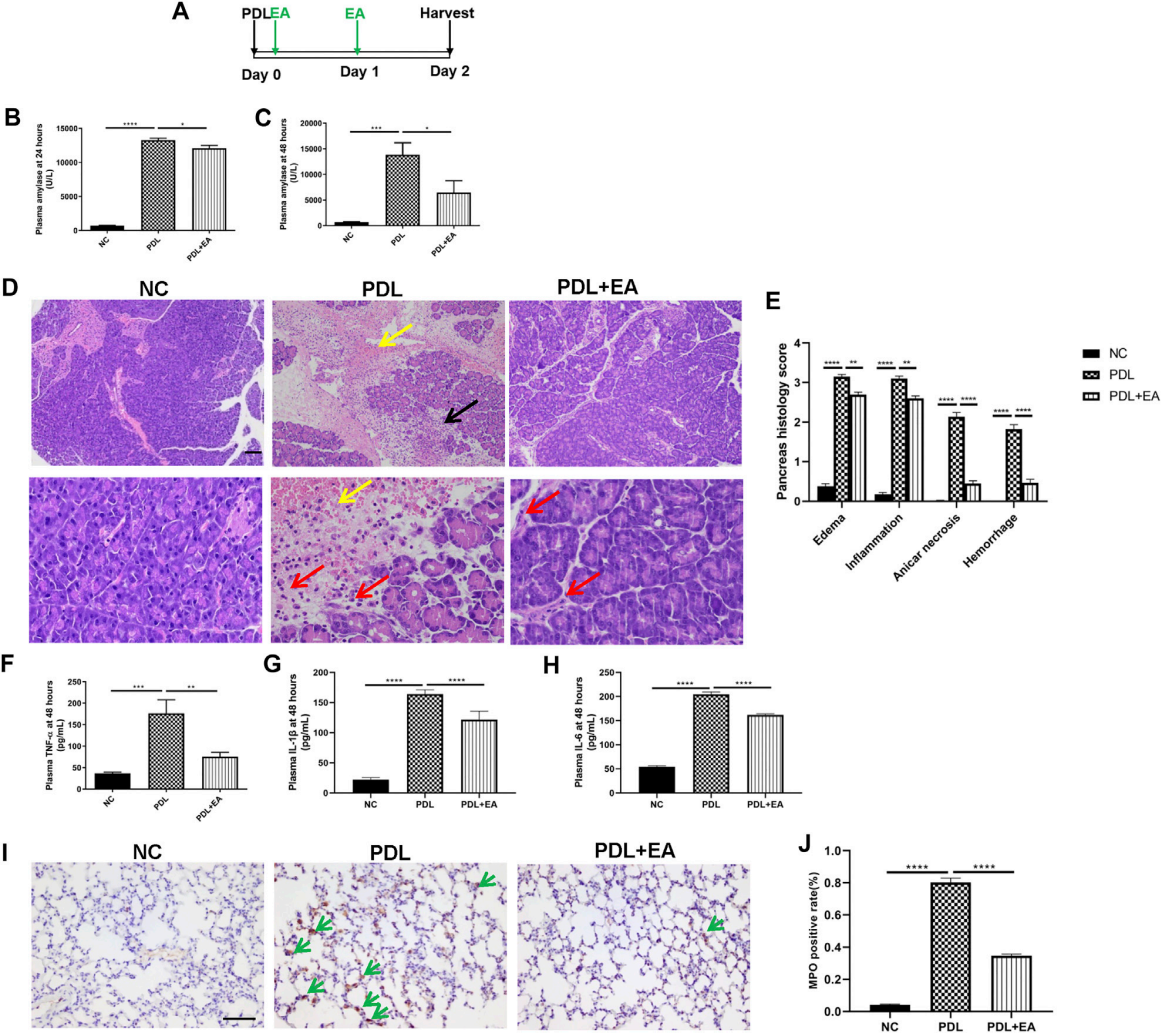
EA alleviates pancreas injury and systemic inflammation in PDL-induced pancreatitis. **(A)** Schematic description of time line. **(B)** Plasma amylase activity at 24 h after PDL operation. **(C)** Plasma amylase activity at 48 h. **(D)** Representative H&E staining of pancreas sections (scale bars: 100 μm). Yellow arrow represents pancreas hemorrhage, black arrow represents pancreas necrosis, and red arrow represents infiltrating cells. **(E)** The pancreas histological score. **(F)** Plasma TNF-*α* at 48 h. **(G)** Plasma IL-1β at 48 h. **(H)** Plasma IL-6 at 48 h. **(I)** Representative immunohistochemical images of MPO expression in the lung (scale bars: 100 μm). Green arrow represents infiltrating MPO^+^ neutrophils. **(J)** Quantitative summary of MPO expression, *n* = 9. Bars show mean ± SEM. *p < 0.05, **p < 0.01, ***p < 0.001, and ****p < 0.0001. NC, normal control; PDL, pancreatic duct ligation; EA, electroacupuncture.

We also confirmed the protective role of EA in the PDL model, which could mimic clinical gallstone caused severe AP associated with significant mortality. EA treatment was applied 30 min and 24 h after operation. Plasma and tissues (pancreas and lung) were harvested at the 48th hour (Figure 6A). Plasma amylase concentrations shut up to 13268 ± 296.2 U/L at 24 h and to 13849 ± 2305 U/L at 48 h after PDL model induction (Figures 6B,C). Massive hemorrhage and intensive leukocytes infiltration were evidenced in the pancreas of PDL mice (Figures 6D,E). Pancreatic injury, as determined by histology scores and amylase activities, was significantly lower in EA-treated mice. EA was also effective in decreasing the systemic inflammation in PDL mice, as evidenced by decreased plasma cytokine levels (Figures 6F–H). Acute lung injury is the most common complication of severe AP. EA additionally protected against lung injury. Immunohistochemical staining of MPO^+^ neutrophils in the lung demonstrated a decreased infiltration in EA-treated mice (Figures 6I,J).

**FIGURE 7:**
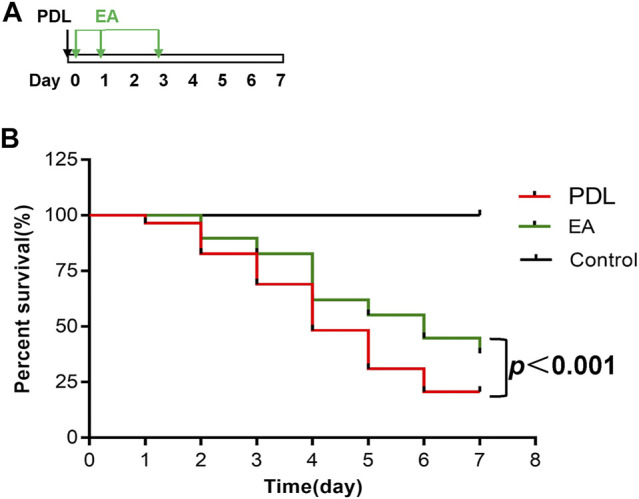
EA prolongs survival of mice with PDL-induced pancreatitis. **(A)** Schematic description of time line. **(B)** The Kaplan–Meier curve presents the survival rate, *n* = 29. PDL, pancreatic duct ligation; EA, electroacupuncture.

For survival studies, EA was applied to the PDL mice 30 min, 1 day, and 3 days after surgery (Figure 7A). EA treatment exerted a significant survival benefit in PDL mice. The median survival days were 4 days in the AP group and 6 days in the EA group. EA-treated mice had significantly lower mortality than sham EA–treated mice (*p* < 0.0001, Figure 7B).

## Discussion

The results of this study showed that EA at Zusanli resulted in decreased severity of experimental AP. The protective effects observed were dependent on the vagus nerve and α7nAChR signaling.

Due to the pain relieving and gastrointestinal motility–promoting functions, acupuncture is popular in AP treatment in Chinese hospitals. Clinical studies, although with doubtful quality, found that EA may be effective for reducing the duration of abdominal pain, TNF-*α* count, and the length of hospital stay in AP patients (Zhu et al., 2015; Zhang et al., 2019). The acupoints used in AP treatment are diverse, including Zusanli (ST-36), Shangjuxu (ST-37), Taichong (ST-25), Zhigou (TE-6) and et al. (Zhang et al., 2019). In the present study, we chose the most popular acupoint, that is, ST-36. To determine the therapeutic role of EA at ST-36, we established AP in mice by caerulein hyperstimulation and PDL. EA was effective in treating local (pancreas), distant (lung), and systemic inflammation in AP. Our results are consistent with those in the study by Guo et al., showing a protective effect of EA in sodium tautocholate–induced AP in rats (Guo et al., 2014).

In AP, the initial inflammatory process leads to migration of monocytes and neutrophils into the pancreas (Dixit et al., 2019). Neutrophils have been proposed to play crucial roles in the early phase of disease development, contributing to activation of trypsinogen and progression to severe AP (Habtezion, 2015). We found that the recruitment of neutrophils was significantly reduced inside the pancreas and lung tissues by EA. Monocytes and macrophages are key inflammatory cells involved in the pathogenesis of AP. The infiltrating macrophages produce inflammatory mediators and cytokines such as TNF-α, IL-1β, and IL-6. These mediators, in turn, recruit more leukocytes and increase the inflammatory response in the pancreas, followed by involvement of distant organs such as the lung (Habtezion, 2015). Our results showed that EA suppressed macrophage infiltration in the pancreas and TNF-α, IL-1β, and IL-6 levels in the plasma. Taken together, these results indicated that EA could exert anti-inflammatory effects by inhibiting neutrophil and macrophage recruitment in AP.

Previous studies have suggested acupuncture at ST-36 can suppress systemic inflammation, mainly or partly *via* activation of vagal efferents. In the lipopolysaccharide (LPS)-induced sepsis mice model, EA at ST-36 (intensity 40 mA, frequency 10 Hz) controlled systemic inflammation through activation of the vagus nerve and increasing dopamine production from the adrenal medulla (Torres-Rosas et al., 2014). Recent research by Shenbin Liu investigated the impact of different intensities at ST-36 on the autonomic pathway in septic mice (Liu et al., 2020). They found 0.5 mA at ST-36 was sufficient to induce c-Fos in hindbrain choline acetyltransferase positive vagal efferent neurons located in dorsal motor nuclei of the vagus. In caerulein hyperstimulation mice, we found EA at ST-36 significantly decreased LF/HF, indicating parasympathetic dominant arose from EA. Furthermore, EA failed to protect against AP in mice subjected to vagotomy. Therefore, the anti-inflammatory effects of EA in our study are vagal nerve dependent.

The vagus nerve controls immune responses and pro-inflammatory cytokine production through the cholinergic anti-inflammatory pathway (Borovikova et al., 2000). Efferent vagus nerve fibers provide a conduit for brain-to-immune communications to control the production of pro-inflammatory cytokines through the release of acetylcholine (Chavan et al., 2017). Several studies have reported that EA at ST-36 increased acetylcholine release. In a rat model of diet-induced obesity, EA at ST-36 increased serum acetylcholine levels (Jie et al., 2018). Research has also reported EA at ST-36 increased acetylcholine esterase in the stomach and the jejunum in rabbits (Niu et al., 2007). The released acetylcholine interacts with α7nAChR, which is expressed on macrophages and monocytes, resulting in the suppression of pro-inflammatory cytokine production (Pavlov and Tracey, 2017). Delineating intracellular mechanisms downstream of α7nAChR have indicated a role for activation of Janus Kinase 2 and the subsequent phosphorylation of a signal transducer and activator of transcription 3, as well as the suppression of nuclear factor-kappa B nuclear translocation (de Jonge et al., 2005). Our results showed that EA resulted in a remarkable increase in the percentage of α7nAChR^+^ macrophages in the pancreatic tissue. Next, a specific α7nAChR antagonist, methyllycaconitine citrate, was found to counteract the ameliorating effects of EA in AP mice. All these findings clearly pointed to a role of α7nAChR in EA regulation of inflammation in AP.

Both invasive and noninvasive techniques of vagus nerve stimulation (VNS) exist. Invasive VNS involves surgical implantation of a programmable pulse generator device in the chest wall and placement of electrodes around the left cervical vagus nerve (Butt et al., 2020). The invasive method is not feasible for patients with acute inflammation. Among the noninvasive techniques, auricular VNS seems to have potential in acute inflammatory diseases (He et al., 2013). The auricular branch of the vagus nerve mainly innervates the external auditory canal and the auricular concha (He et al., 2012). Functional magnetic resonance imaging of the brain has demonstrated that auricular VNS can stimulate brain areas consistent with the contemporaneously accepted understanding of central vagal projections (Frangos et al., 2015). Auricular VNS was shown to be efficient in reducing systemic inflammation in mice with lethal endotoxemia or polymicrobial sepsis (Butt et al., 2020). However, the evidence of auricular VNS in AP is lacking.

Some limitations should be acknowledged. First, LF/HF has received some criticism as a measure of sympathovagal balance (von Rosenberg et al., 2017). Frequency domain analysis of HRV reveals two or more peaks, a LF peak (<0.15 Hz) and a HF peak (>0.15 Hz), which are assumed to correspond to cardiac sympathetic and parasympathetic nervous activity. However, some research studies found this assumption oversimplifies the complex of nonlinear interactions between the sympathetic and the parasympathetic divisions of the autonomic nervous system (Billman, 2013). A more accurate evaluation of parasympathetic tone, such as the c-Fos expression in the dorsal motor nucleus of the vagus, is needed to verify our findings in further explorations. Second, vagotomy was performed by transection of the left cervical vagus nerve, given the safety concerns. The right vagus nerve innervates predominantly the sinus node, and right cervical vagotomy might adversely affect the cardiovascular system (Michael and Robert, 2014). The effect of the right vagus nerve on pancreatitis remains to be explored. Third, α7nAChR is expressed on neurons and immune cells (Báez-Pagán et al., 2015). Although our data imply a crucial role for α7nAChR in the anti-inflammatory effects of EA, the cell type (neurons or macrophages) carrying the functional α7nAChR remains to be elucidated in further studies.

In conclusion, we found EA at ST-36 alleviated the severity of AP through activation of the vagus nerve–based cholinergic anti-inflammatory pathway.

## Data Availability

The original contributions presented in the study are included in the article.

## Abbreviations

AP, acute pancreatitis; EA, electroacupuncture; HRV, heart rate variability; i.p., intraperitoneal injections; LF/HF, low frequency/high frequency; MLA, methyllycaconitine citrate; NC, normal control; PDL, pancreatic duct ligation; VGX, vagotomy; VNS, vagus nerve stimulation; α7nAChR, α7 nicotinic acetylcholine receptor
